# Perirenal fat stranding as a predictor of disease progression after radical nephroureterectomy for renal pelvic urothelial carcinoma: a retrospective study

**DOI:** 10.1007/s12672-023-00741-z

**Published:** 2023-07-03

**Authors:** Masato Yanagi, Mika Terasaki, Tomonari Kiriyama, Yasuhiro Terasaki, Jun Akatsuka, Yuki Endo, Taiji Nishimura, Akira Shimizu, Yukihiro Kondo

**Affiliations:** 1 Department of Urology, Nippon Medical School Hospital, 1-1-5, Sendagi, Bunkyo-ku, Tokyo, 113-8603 Japan; 2 Department of Analytic Human Pathology, Nippon Medical School Hospital, 1-1-5, Sendagi, Bunkyo-ku, Tokyo, 113-8603 Japan; 3 Department of Radiology, Nippon Medical School Hospital, 1-1-5, Sendagi, Bunkyo-ku, Tokyo, 113-8603 Japan

**Keywords:** Urothelial carcinoma, Renal pelvic urothelial carcinoma, Hydronephrosis, Perirenal fat stranding, Nephroureterectomy, Progression

## Abstract

**Background:**

To investigate the impact of Perirenal fat stranding (PRFS) on progression after radical nephroureterectomy (RNU) for renal pelvic urothelial carcinoma (RPUC) without hydronephrosis and to reveal the pathological findings of PRFS.

**Methods:**

Clinicopathological data, including computed tomography (CT) findings of the ipsilateral PRFS, were collected from the medical records of 56 patients treated with RNU for RPUC without hydronephrosis between 2011 and 2021 at our institution. PRFS on CT was classified as either low or high PRFS. The impact of PRFS on progression-free survival (PFS) after RNU was analyzed using the Kaplan–Meier method and log-rank test. In addition, specimens including sufficient perirenal fat from patients with low and with high PRFS were pathologically analyzed. Immunohistochemical analysis of CD68, CD163, CD3, and CD20 was also performed.

**Results:**

Of the 56 patients, 31(55.4%) and 25 (44.6%) patients were classified as having low and high PRFS, respectively. Within a median follow-up of 40.6 months postoperatively, 11 (19.6%) patients showed disease progression. The Kaplan–Meier method and log-rank test revealed that patients with high PRFS had significantly lower PFS rates than those with low PRFS (3-year PFS 69.8% vs 93.3%; p = 0.0393). Pathological analysis revealed that high PRFS specimens (n = 3 patients) contained more fibrous strictures in perirenal fat than low PRFS specimens (n = 3 patients). In addition, M2 macrophages (CD163 +) infiltrating fibrous tissue in perirenal area were observed in all patients with high PRFS group.

**Conclusions:**

PRFS of RPUC without hydronephrosis consists of collagenous fibers with M2 macrophages. The presence of ipsilateral high PRFS might be a preoperative risk factor for progression after RNU for RPUC patients without hydronephrosis. Prospective studies with large cohorts are required in the future.

**Supplementary Information:**

The online version contains supplementary material available at 10.1007/s12672-023-00741-z.

## Introduction

Upper tract urothelial carcinoma (UTUC) is a relatively uncommon disease, accounting for only 5–10% of all urothelial carcinomas [1]. Radical nephroureterectomy (RNU) is the gold standard treatment for UTUC without metastasis. However, local or metastatic recurrence after RNU for UTUC frequently occurs, with an incidence rate of approximately 24–28% [2, 3]. Several studies have investigated the risk factors for local or metastatic recurrence after RNU and identified hydronephrosis on preoperative computed tomography (CT) [4, 5], lymphovascular invasion (LVI) [6, 7], and positive surgical margin [8]. Recently, perirenal fat stranding (PRFS) has also been reported as a risk factor for recurrence after RNU for ureteral urothelial carcinoma (UUC) with hydronephrosis [9].

PRFS is often recognized as a linear or curvilinear area of soft-tissue attenuation in the perirenal fat space on CT. There have been reports of PRFS due to pyelonephritis and ureteral stones with hydronephrosis in benign diseases [10, 11] and reports of PRFS due to UUC with hydronephrosis in cancers [9]. However, PRFS in renal pelvic urothelial carcinoma (RPUC) without hydronephrosis has not been reported. In addition, the mechanism of appearance and pathological findings of PRFS remains unknown. Fortunately, some preserved specimens obtained by RNU for RPUC contain sufficient perirenal fat. Therefore, the pathological analysis of PRFS associated with RPUC is possible.

## Materials and methods

### Study design and patients

**Fig. 1:**
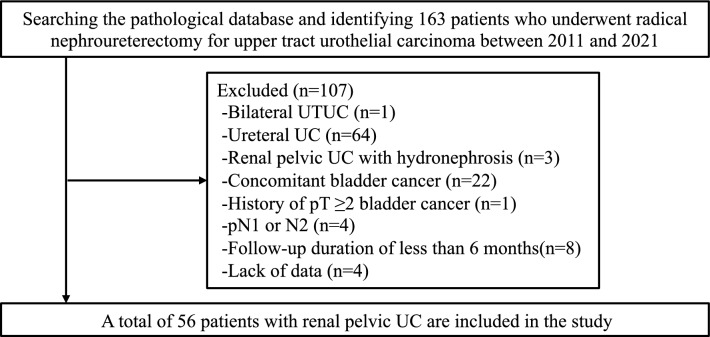
Patient selection flowchart. UTUC, upper tract urothelial carcinoma

This retrospective study aimed to investigate the impact of PRFS on progression after RNU for RPUC without hydronephrosis and to reveal the pathological characteristics of PRFS. In total, 163 patients who underwent RNU for UTUC at the Nippon Medical School Hospital between 2011 and 2021 were evaluated. Among them, patients with bilateral UTUC, UUC, RPUC with hydronephrosis, concomitant bladder cancer, history of pT ≥ 2 bladder cancer, pN1, or pN2, and a follow-up duration of < 6 months or insufficient data were excluded. Finally, 56 patients with RPUC but without hydronephrosis were included in the present study (Fig. 1).

### Data collection

Demographic and clinicopathological information, including age, sex, body mass index (BMI), history of pT < 2 bladder cancer, laterality, cT stage, ipsilateral PRFS on preoperative CT, preoperative urine cytology, history of diagnostic ureteroscopic biopsy, surgical method of RNU, multifocality, tumor size, pT stage, grade, LVI, infiltrative growth (INF), surgical margin, history of adjuvant systemic chemotherapy, and outcomes, were collected from the medical records. Tumor grading was according to the 2004 World Organization Classification, and staging was according to the 2002 American Joint Committee of Cancer tumor-node-metastasis classification.

### Surgical procedure

In our institution, ureteroscopic biopsy is performed by endourology experts to avoid complications, including ureteral injury and pyelonephritis due to increased intrapelvic pressure. In addition, only two cold punch biopsies are performed on the tumor. Retroperitoneoscopic RNU or open peritoneal RNU for RPUC is performed in our institution. In retroperitoneoscopic RNU, a small iliac incision or lower abdominal midline incision is made to perform bladder cuff resection using an extravesical approach. In open RNU, bladder cuff resection is performed using an extravesical approach. Lymphadenectomy is not performed in the retroperitoneoscopic RNU group. Lymphadenectomy is performed for clinical N1 or N2 patients using open surgery.

### Adjuvant therapy and follow-up methods

In our institution, preoperative systemic chemotherapy and adjuvant intravesical chemotherapy are not administered. Four courses of adjuvant systemic chemotherapy (ASC), such as the gemcitabine/cisplatin or gemcitabine/carboplatin regimen, is administered to select patients with pT ≥ 2. Patients with an estimated glomerular filtration rate of < 30 mL/min/1.73m^2^ undergo ASC with gemcitabine/carboplatin regimen, and other patients undergo ASC with gemcitabine/cisplatin regimen. Postoperative follow-up generally includes urine analysis, urine cytology, blood tests, cystoscopy, and CT every 3 months for 2 years and every 6 months thereafter. In this study, progression was defined as radiologically diagnosed local or metastatic recurrence.

### Imaging techniques and image analysis

Abdominal CT images were obtained with various scanners inside and outside the hospital using 64- to 320-channel multidetector CTs. There is no uniform scan protocol; however, an example of an average scan protocol is provided below. Non-contrast-enhanced acquisition of the entire abdomen and pelvis is followed by triphasic dynamic CT if available. A bolus of 80–150 ml of iodine contrast media (300–370 mg iodine/mL) was administered via cubital vein at 2-3 ml/s according to patients’ body weight. The scan timings of the dynamic CT are as follows: corticomedullary phase, 30–40 s after the injection; nephrographic phase, approximately 90–100 s; and excretory delayed phase: approximately 300 s. The scanning parameters are as follows: tube voltage, 120 keV; auto mA modulation, FOV 300–400 mm; gantry rotation time, 0.4–0.5; collimation, 40–80 mm; and pitch, 0.9–1.0.

**Fig. 2:**
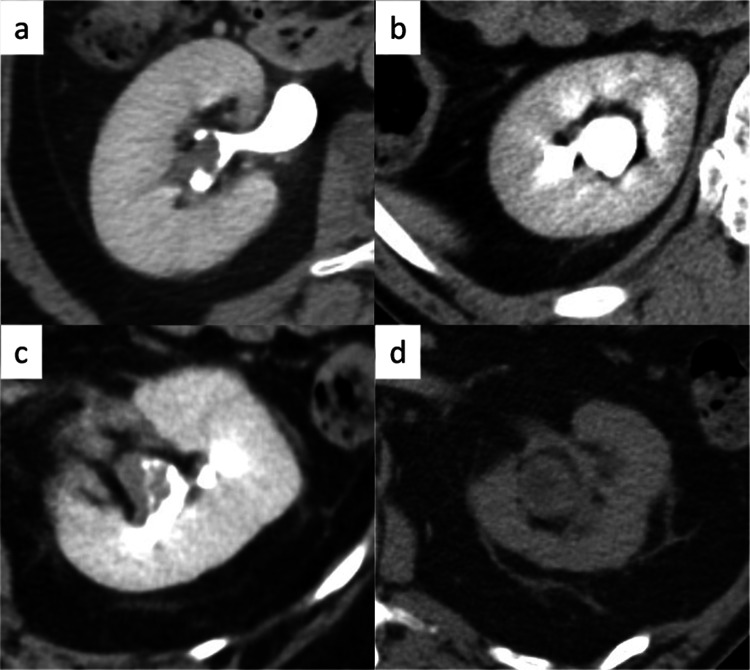
Finding of PRFS on the excretory phase of CT. The PRFS is graded according to its degree. **a** No PRFS. **b** Mild PRFS. **c** Moderate PRFS. **d** Severe PRFS. No and mild PRFS are defined as low PRFS. Moderate and severe PRFS are defined as high PRFS. *PRFS* perirenal fat stranding

In this study, a staff urologist (MY) with 13 years of experience collected data from contrast-enhanced CT scans performed before ureteroscopic biopsy and within 3 months before surgery. PRFS was categorized according to the method used for benign diseases by Kim et al. [12]. Cases without PRFS and those with a few thin strands were defined as having low PRFS. Cases with more and/or thicker stranding than those with low PRFS were defined as having a high PRFS (Fig. 2). A staff urologist (JA) with 14 years of experience in urological oncology and a staff radiologist (TK) with 20 years of experience in genitourinary imaging independently analyzed the CT images. Both assessors were blinded to the clinicopathological information and outcomes of the patients. Diagnostic discrepancies were resolved by consensus.

### Pathological analysis of PRFS

Of the specimens that included sufficient perirenal fat, those of patients without PRFS and those of patients with PRFS all around the kidney were pathologically analyzed. Formalin-fixed paraffin-embedded tissue sections were cut into 4-μm-thick slices and stained with hematoxylin and eosin (H&E), Elastica Masson-Goldner (EMG) staining. Immunohistochemical analysis of CD68, CD163, CD3, and CD20 was also performed. The antibodies and staining conditions used in the present study are summarized in Supplementary Table1. The polymer immunocomplex system consisting of peroxidase/3,3ʹ-diaminobe (DAB) + (N-Histofine Simple Stain MAX PO (ready-to-use), Nichirei Biosciences Inc., Tokyo, Japan) for rabbit/mouse primary antibodies, was used for secondary antibody treatment and protein visualization. Two staff pathologists with more than 20 years of experience (MT and AS) who were blinded to the patients’ radiological findings and clinical outcomes analyzed the pathological findings.

### Statistical analysis

Categorical variables were compared using Fisher’s exact test and continuous variables using the t-test or Mann–Whitney U test, depending on the results of the one-sample Kolmogorov–Smirnov test. Survival curves were generated using the Kaplan–Meier method, and survival differences between the low PRFS group and the high PRFS group were evaluated using the log-rank test. In addition, the associations between high PRFS and progression after RNU and progression within 2 years after RNU were analyzed using inverse propensity-weighted (IPW) adjusted logistic regression analysis. Variables adjusted for PRFS included multiple tumors, tumor size ≥ 3 cm, pT ≥ 3, cancer grade 3, positive LVI, INF b, c, positive surgical margin, and ASC. All statistical analyses were performed using IBM SPSS Statistics version 27 (IBM, Armonk, NY, USA). A p-value of < 0.05 was considered statistically significant.

## Results

Based on preoperative CT analysis of ipsilateral PRFS of the 56 patients, 31 (55.4%) and 25 (44.6%) patients were categorized as having low and high PRFS, respectively. Open RNU with lymphonodectomy was performed in 5 (8.9%) patients (Table 1). All five patients who underwent lymphonodectomy were diagnosed with N0. Overall, 40 (71.4%), 12 (21.4%), 40 (71.4%), and 2 (3.6%) patients were diagnosed with grade 3, LVI positive, INF ≥ b, and surgical margin positive disease, respectively. In addition, the surgical margins of the bladder were negative in all the patients.

**Table 1:** Patient characteristics

Variables		n = 56 (%)
Age (years)	Median (IQR 25–75)	77 (70–82)
Sex	Male/female	38 (67.9)/18 (32.1)
BMI (kg/m^2^)	Median (IQR 25–75)	22.4 (20.9–24.4)
History of pT < 2 bladder cancer	Yes/no	9 (16.1)/47 (83.9)
Laterality	Left /right	28 (50.0) /28 (50.0)
cT	≤ 1/2/ ≥ 3	28 (50.0)/15 (26.8)/13 (23.2)
PRFS	Low/high	31 (55.4)/25 (44.6)
Urine cytology	Positive/negative	21 (37.5)/35 (62.5)
Diagnostic ureteroscopic biopsy	Yes/no	15 (26.8)/41 (73.2)
Surgical method	Laparoscopic/ open	51(91.1)/ 5 (8.9)
ASC	Yes/no	8 (14.3)/48 (86.7)

*IQR* interquartile range, *BMI* body mass index, *PRFS* perirenal fat stranding, *ASC* adjuvant systemic chemotherapy

Table 2 shows a comparison of patient characteristics between those with high PRFS and those with low PRFS. The high PRFS group was significantly older (p = 0.0094) and included a significantly larger proportion of males (p = 0.0037), patients with tumors on the left side (p = 0.0066), and patients with multiple tumors (p = 0.0168) than their counterparts. However, there was no significant difference in pathological characteristics such as cancer grade, LVI, INF, and surgical margin between the high PRFS and the low PRFS groups.

**Table 2:** Comparison of patients with low PRFS and patients with high PRFS

Variables	Low PRFS n = 31	High PRFS n = 25	p Value
Age (years), median (IQR 25–75)	75 (69–80)	81 (76–85)	0.0094^*^
Male	16 (51.6)	22 (88.0)	0.0037^*^
BMI (kg/m^2^), median (IQR 25–75)	21.9 (20.8–24.0)	23.1 (20.9–25.2)	0.2839
History of pT < 2 bladder cancer	5 (13.5)	4 (21.1)	0.4703
Left	10 (32.3)	18 (72.0)	0.0066^*^
cT ≥ 3	5 (16.1)	7 (28.0)	0.3376
Positive urine cytology	12(38.7)	9 (36.0)	1.0000
Multiple tumors	1 (3.2)	7 (28.0)	0.0168^*^
Tumor size ≥ 3 cm	8 (25.8)	10 (40.0)	0.3884
pT ≥ 3	7 (22.6)	10 (40.0)	0.2425
Cancer grade 3	24 (77.4)	16 (64.0)	0.3739
LVI ( +)	8 (25.8)	4 (16.0)	0.5163
INF b, c	21 (67.7)	19 (76.0)	0.5625
Surgical margin ( +)	2 (6.5)	0 (0.0)	0.4968
ASC	4 (12.9)	4 (16.0)	0.7426
Local recurrence	1 (3.2)	0 (0)	1.0000
Distant metastasis	3 (9.7)	7 (28.0)	0.0921

*PRFS* perirenal fat stranding, *IQR* interquartile range, *BMI* body mass index, *LVI* lymphovascular invasion, *INF* infiltrative growth, *ASC* adjuvant systemic chemotherapy

^*^p < 0.05

**Fig. 3:**
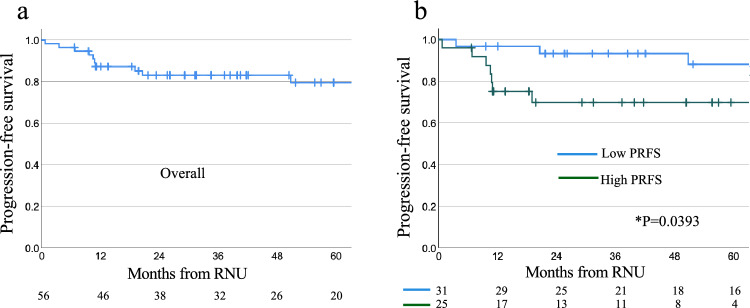
Kaplan–Meier curves of PFS after RNU in the 56 RPUC patients. **a** PFS in 56 patients. **b** Comparison of PFS between patients with low and with high PRFS. *PFS* progression-free survival, *PRFS* perirenal fat stranding

Within a median follow-up of 40.6 months postoperatively, 11 (19.6%) patients experienced progression. Of these patients, five had lung metastases, two had distant lymph node metastases, one had lung and lymph node metastases, one had lung and pancreatic metastases, one had liver metastases, and one had local recurrence. The 1-year and 3-year progression-free survival (PFS) rates in the overall population were 87.2% and 83.0%, respectively (Fig. 3a). The Kaplan–Meier curve and log-rank test revealed that the high PRFS group had significantly lower PFS than the low PRFS group (3-year PFS: 69.8% vs 93.3%; p = 0.0393) (Fig. 3b). Within a median follow-up of 42.1 months postoperatively, 1 (1.8%) patient with high PRFS died from the disease. Table 3 demonstrates how high PRFS is associated with progression after RNU and progression within 2 years after RNU. Progression within 2 years after RNU was significantly higher in the high PRFS group than in the low PRFS group in both the unadjusted model [odds ratio (OR), 9.47, 95% confidence interval (CI) 1.06–84.96, p = 0.045] and the IPW model (OR17.79, 96% CI 1.74–182.23, p = 0.015).

**Table 3:** Associations between high PRFS and progression after RNU

		Progression after RNU			Progression within 2 years after RNU		
Full cohort		OR	95% CI	P value	OR	95% CI	P value
Unadjusted model	n = 56	2.62	0.67–10.29	0.166	9.47	1.06–84.96	0.045^*^
IPW model	n = 111	3.64	0.779–16.98	0.100	17.79	1.74–182.23	0.015^*^

*PRFS* perirenal fat stranding, *RNU* radical nephroureterectomy, *OR* odds ratio, *CI* confidence interval, *IPW* inverse propensity-weighted

^*^p < 0.05

**Fig. 4:**
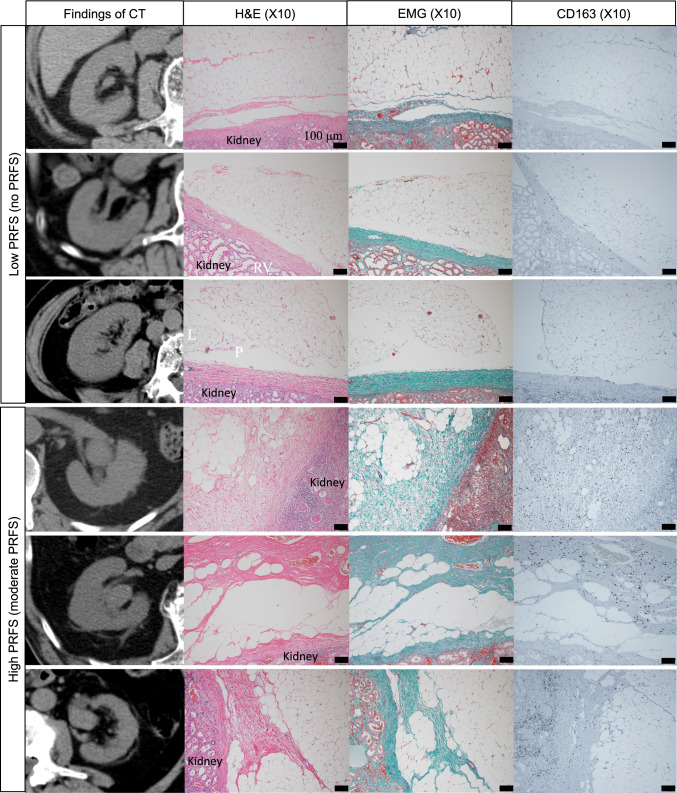
H&E, EMG, CD163 staining for perirenal fat of 3 patients without PRFS (low PRFS) and 3 patients with moderate PRFS (high PRFS). *PRFS*, perirenal fat stranding

In pathological analysis, patients without paraffin block and patients without sufficient fat were excluded (Supplementally Fig. 1). Finally, 3 specimens of patients without PRFS and 3 specimens of patients with PRFS all around the kidney were pathologically analyzed. Figure 4 demonstrates the CT and pathological findings of the three patients with low PRFS and the three patients with high PRFS. H&E and EMG staining of the specimens revealed that the high PRFS specimens contained more fibrous strictures in the perirenal fat than did the low PRFS specimens (Fig. 4). Immunohistochemical analysis revealed that the perirenal areas of all cases were not positive for CD3 and CD20. (Supplementally Fig. 2). On the other hand, M2 macrophages (CD68 + and CD163 +) infiltrating fibrous tissue in perirenal area were observed in all cases of the high PRFS group (Fig. 4).

## Discussion

There have been several studies on PRFS with hydronephrosis [9–15]. In general, the occurrence of PRFS with hydronephrosis can be explained by the following two reasons. The first possible explanation is that inflammation and bridging septa fibrosis may result from extravasated urine [9, 13]. When the ureter becomes obstructed, increased intrarenal pelvic pressure results in a microscopic rupture at the calyceal fornix, which is the area of least resistance, and pyelosinus backflow of urine may occur. Subsequently, extravasated urine from the renal hilum enters the perirenal space and infiltrates the bridging septa. The second possible explanation is that thickening of the bridging septa may result from lymphatic dilation and extravasation [14, 15]. When the dilated renal pelvis compresses the hilar lymphatics, the lymph flow diverts to the perinephric lymphatics, which run along the fibrous septa of the perinephric space.

The present study focused on PRFS in patients without hydronephrosis. Han et al. reported that bilateral PRFS on CT often appears in older male patients without hydronephrosis [16]. They hypothesized that a long-term intermittent increase in intrarenal pressure due to chronic obstruction of the bladder outlet from lower urinary tract symptoms causes recurrent extravasation of urine, resulting in low-grade inflammation and bridging septa fibrosis, which is shown as PRFS on CT. In the present study, most of the patients with PRFS had bilateral PRFS. The results of this study suggested that PRFS in patients with RPUC without hydronephrosis was not solely caused by RPUC. In general, elderly men often experience lower urinary tract symptoms over the long term. In this study, the high PRFS group was significantly older (p = 0.0094) and included significantly more male patients (p = 0.0037) than the low PRFS group. Therefore, the PRFS in our cohort was probably associated with long-term intermittent increases in intrarenal pelvic pressure due to lower urinary tract symptoms. However, it was difficult to review patient information regarding the duration and severity of lower urinary tract symptoms. Further prospective studies on the association between lower urinary tract symptoms and PRFS in RPUC patients are required.

In pathological analysis in the present study, the specimens with high PRFS contained fibrous strictures in the perirenal fat (Fig. 4). In the specimens with high PRFS, neither dissemination of tumor cells in perirenal fat nor lymphocyte invasion in perirenal fat was confirmed. However, M2 macrophages infiltrating fibrous tissue in perirenal area were observed in those specimens (Fig. 4). In addition, PRFS was not enhanced in the excretory phase of contrast-enhanced CT (Fig. 2a–d). This suggested that in RPUC, PRFS without hydronephrosis are fibrous strictures with M2 macrophages, not lymph or urine. To our best knowledge, this is the first study to reveal the pathological characteristics of PRFS.

The Japanese Clinical Oncology Group reported that a rapid increase in intrarenal pelvic pressure due to acute urinary tract obstruction contributed to cancer progression [17]. They evaluated 664 patients with non-metastatic UTUC who underwent RNU with intraoperative ureteral ligation and found significantly higher cancer-specific mortality in patients who underwent ureteral ligation before renal vascular ligation than in those who underwent ureteral ligation after renal vascular ligation. Early ureteral ligation resulted in a rapid increase in the intrarenal pelvic pressure. Thus, they hypothesized that urine-containing tumor cells in the upper urinary tract leaked into lymphovascular vessels and/or the retroperitoneal space secondary to iatrogenic urinary obstruction by early ureteral ligation, which resulted in distant metastasis or local recurrence. In the present study, patients with a high PRFS had significantly worse PFS than those with a low PRFS. The results supported that in RPUC, PRFS without hydronephrosis reflected mild inflammation and fibrosis caused by urinary extravasation due to long-term intermittent high intrarenal pelvic pressure. Further, long-term intermittent high intrarenal pelvic pressure caused PRFS and contributed to cancer progression. In general, M2 macrophage is associated with the growth of fibrous tissue and tumor growth/development [18]. In this study, M2 macrophages infiltrating fibrous tissue in perirenal area were observed in all cases with high PRFS. One of the possible explanations is that the tumors destroy the surrounding tissue, resulting in the induction of M2 macrophages, which cause fibrosis around the kidney. However, the mechanism of the appearance of M2 macrophages in the perirenal area distant from the tumor remains unclear. From the results of the present study, the appearance of PRFS may be associated with high intrarenal pelvic pressure and RPUC. Further studies about mechanism of appearance of PRFS are required.

The present study has several limitations. First, the sample size was small owing to the rarity of renal pelvic cancer and the single-center study design. Multivariate analysis was precluded. Moreover, the pathological differences between the low PRFS group and the high PRFS group have large discrepancies. To overcome this limitation, we performed IPW adjusted logistic regression analysis. Second, there might be several biases because of the retrospective nature of the study. Especially, the influence of ASC cannot be completely excluded. Further prospective studies with larger cohorts from other institutions with multivariate analyses are required. Third, there were few specimens with sufficient perirenal fat in the present study because resection without perirenal fat is allowed in RNU and old specimens had been discarded. Future studies with pathological analyses in a large cohort of patients who undergo RNU with resection of sufficient perirenal fat are required. Fourth, the generalizability of the results to PRFS of other diseases is unclear. This study only analyzed PRFS in RPUC without hydronephrosis. Further studies on the pathological findings of PRFS in other diseases are required. Finally, lymphadenectomy is generally recommended for pathological T ≥ 2 UTUC. However, lymphonodectomy was not performed in most patients in the present study. This is because there are several discrepancies between the clinical and pathological T stages, and there are technical issues with retroperitoneoscopic lymphadenectomy. The absence of lymphadenectomy might have affected the PFS of the cohort in the present study. Lymphadenectomy for clinical T ≥ 2 UTUC is required.

## Conclusions

The PRFS of RPUC without hydronephrosis is constructed with collagenous fibers with M2 macrophages. The presence of ipsilateral high PRFS might be a preoperative risk factor for progression after RNU for patients with RPUC without hydronephrosis. Prospective studies with large cohorts are required in the future.

## Supplementary Information

Supplementary file1

Supplementary file2

Supplementary file3

## Data Availability

The datasets used and/or analyzed during the current study are available from the corresponding author on reasonable request.

## Abbreviations

ASC: Adjuvant systemic chemotherapy
BMI: Body mass index
CI: Confidence interval
CT: Computed tomography
EMG: Elastica Masson-Goldner
H&E: Hematoxylin and eosin
INF: Infiltrative growth
IPW: Inverse propensity-weighted
LVI: Lymphovascular invasion
OR: Odds ratio
PFS: Progression-free survival
PRFS: Perirenal fat stranding
RNU: Radical nephroureterectomy
RPUC: Renal pelvic urothelial carcinoma
UTUC: Upper tract urothelial carcinoma
UUC: Ureteral urothelial carcinoma
