# Supplementary figures and images for: Perirenal fat stranding as a predictor of disease progression after radical nephroureterectomy for renal pelvic urothelial carcinoma: a retrospective study
Source: Discov Oncol. 2023 Jul 3;14:122. doi: 10.1007/s12672-023-00741-z (PMC10317934; doi:10.1007/s12672-023-00741-z)

**Supplementally Figure 1.** Patient selection flowchart of pathological analysis

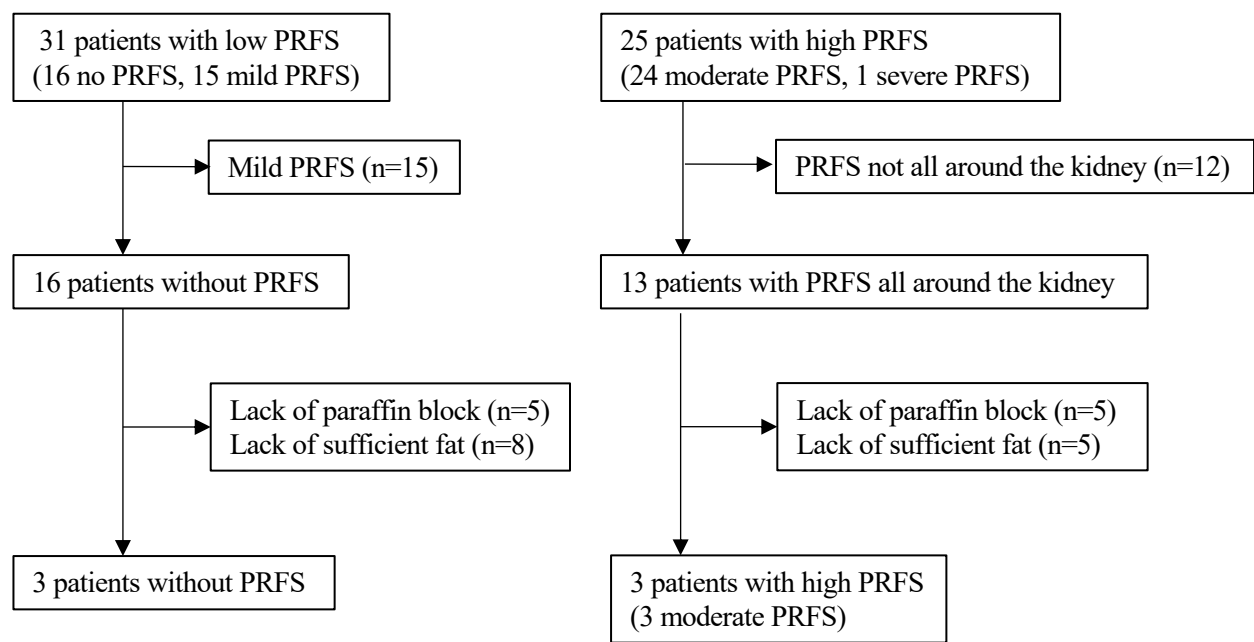

Supplement: Supplementary file 1 — Supplementary file1 [file 12672_2023_741_MOESM1_ESM.pdf]
