# Supplementary material for: Perirenal fat stranding as a predictor of disease progression after radical nephroureterectomy for renal pelvic urothelial carcinoma: a retrospective study
Source: Discov Oncol. 2023 Jul 3;14:122. doi: 10.1007/s12672-023-00741-z (PMC10317934; doi:10.1007/s12672-023-00741-z)
Supplement: Supplementary file 2 — Supplementary file2 [file 12672_2023_741_MOESM2_ESM.pdf]

Supplementally Figure 2. CD68, CD3 and CD20 staining for perirenal fat of 3 patients without ipsilateral PRFS (low PRFS) and 3 patients with ipsilateral moderate PRFS (high PRFS).

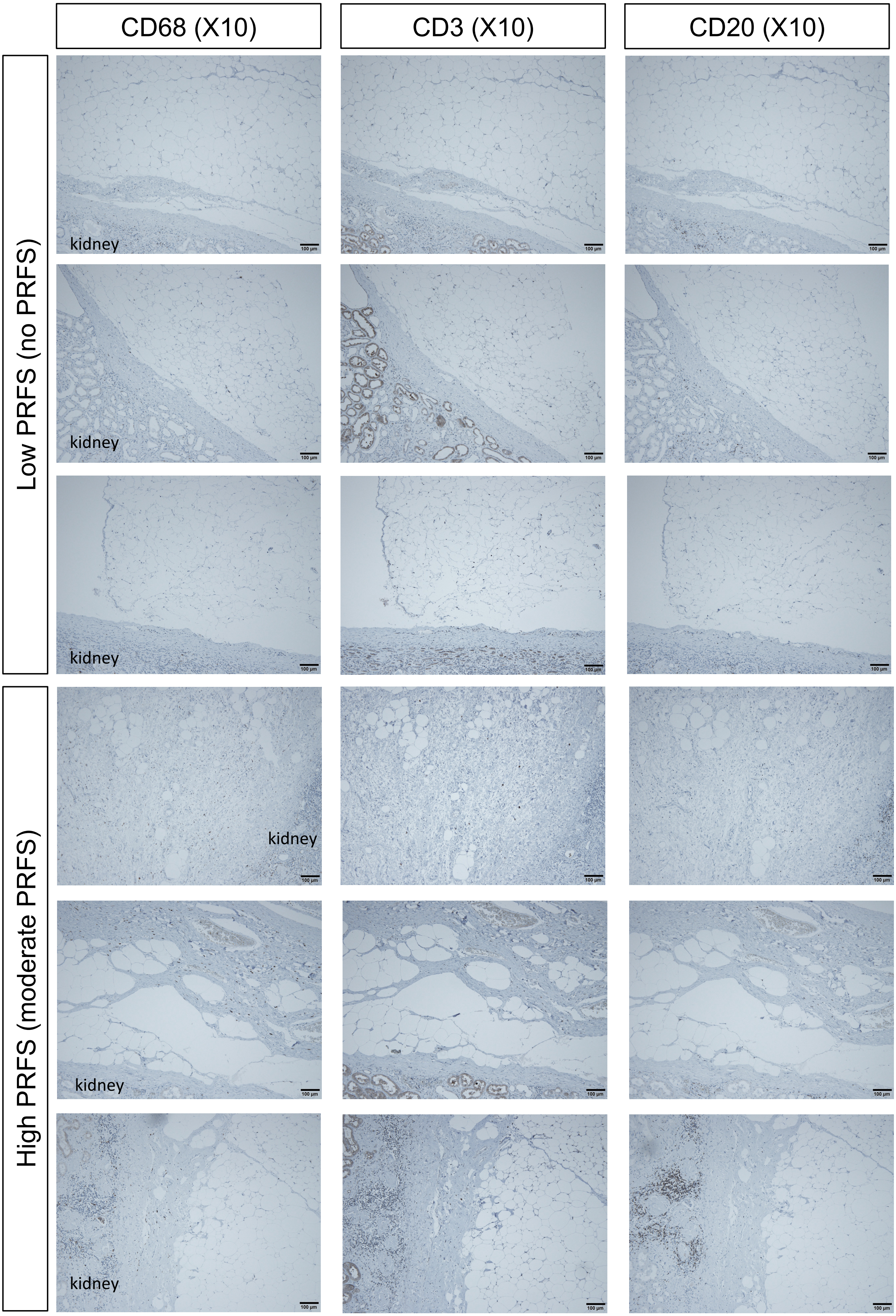

PRFS, perirenal fat stranding
