# Supplementary material for: Perirenal fat stranding as a predictor of disease progression after radical nephroureterectomy for renal pelvic urothelial carcinoma: a retrospective study
Source: Discov Oncol. 2023 Jul 3;14:122. doi: 10.1007/s12672-023-00741-z (PMC10317934; doi:10.1007/s12672-023-00741-z)
Supplement: Supplementary file 3 — Supplementary file3 [file 12672_2023_741_MOESM3_ESM.pdf]

Supplementary Table 1. Antibodies and staining conditions used in this study

Antibody	Animal	Clonality	Clone	Dilution	Antigen retrieval	Vendor
CD68	Mouse	Monoclonal	KP-1	1:500	CB (pH 6); heating in an autoclave for 20 min	DAKO, Santa Clara, CA, USA
CD163	Mouse	Monoclonal	10D6	1:100	CB (pH 6); heating in an autoclave for 20 min	Novocastra, Leica Biosystems, Nussloch, Germany
CD3	Rabbit	Polyclonal		1:500	CB (pH 6); heating in an autoclave for 20 min	DAKO, Santa Clara, CA, USA
CD20	Mouse	Monoclonal	L26	1:1000	CB (pH 6); heating in an autoclave for 20 min	DAKO, Santa Clara, CA, USA
